# Worked examples in the classroom

**DOI:** 10.1007/s40037-015-0226-4

**Published:** 2015-10-26

**Authors:** Jeroen J.G. van Merrienboer

**Affiliations:** School of Health Professions Education, Maastricht University, PO Box 616, 6200 Maastricht, MD The Netherlands

In Cognitive Load Theory (CLT), the kind of schema used in the study by Blissett, Goldszmidt and Sibbald [1] is typically called a Systematic Approach to Problem solving, abbreviated as SAP. A SAP provides the phases to go through when successfully solving a problem, the decisions that must be made by the task performer for entering a particular phase [2], and the rules-of-thumb that might help to successfully complete each phase. Typically, a SAP is combined with worked examples that illustrate the use of the SAP for solving particular problems or, in this study, diagnosing concrete cases. In fact, such worked examples were also provided in the schema-based lecture reported in this article, as the authors state that ‘…murmurs were presented [*by the teacher*] according to timing and location using an adaptation of a published schema [*i.e., the SAP*]… For example, when systolic murmurs at the base of the heart were described, a differential diagnosis of aortic stenosis, atrial septal defect and hypertrophic cardiomyopathy was presented’ (*italics* added). Thus, this study is not only about the effects of the presentation of a schema or SAP, but also about the effects of worked examples given by the teacher to illustrate the use of this SAP.

The results of the presented study are in line with the expectations and provide support to CLT. However, the authors’ claim that the results contribute to the ‘limited’ support for the application of CLT in the classroom deserves the necessary differentiations, especially when we realize that the effects are probably not only due to the presented schema or SAP, but also to the worked examples presented by the teacher. The worked-examples principle is one of the most extensively studied principles of CLT, and there is overwhelming evidence for its effectiveness when teaching novice learners (in contrast to teaching more experienced learners, for whom problem solving might be superior to example study). Several review studies [3] have shown the power of teaching with SAPs and worked examples, and these reviews also include numerous experiments that were conducted in ecologically valid settings. One old but impressive example is reported by Zhu and Simon [4]: They found in a series of long-term studies that worked examples could replace conventional classroom teaching. In one study, they found that a three-year mathematics course was completed in two years by emphasizing worked examples.

Finally, it is important to note that the study by Blissett, Goldszmidt and Sibbald focuses on the presentation of information (i.e., a SAP in combination with worked examples) in a traditional lecture. Although the proper presentation of information is important for facilitating learning, we should never forget that well-designed instruction minimally requires information presentation, practice and feedback. CLT includes not only principles for the presentation of information, but also for the design of practice and feedback [5]. In my opinion, a more integrative approach where information presentation, practice as well as feedback provision are based on CLT will even show larger positive effects on learning, also in ecologically valid settings.
